# Body composition and the monitoring of non-communicable chronic disease risk

**DOI:** 10.1017/gheg.2016.9

**Published:** 2016-10-21

**Authors:** J. C. K. Wells, M. K. Shirley

**Affiliations:** Childhood Nutrition Research Centre, UCL Institute of Child Health, 30 Guilford Street, London WC1N 1EH, UK

**Keywords:** Body composition, chronic disease, non-communicable disease

## Abstract

There is a need for simple proxies of health status, in order to improve monitoring of chronic disease risk within and between populations, and to assess the efficacy of public health interventions as well as clinical management. This review discusses how, building on recent research findings, body composition outcomes may contribute to this effort. Traditionally, body mass index has been widely used as the primary index of nutritional status in children and adults, but it has several limitations. We propose that combining information on two generic traits, indexing both the ‘metabolic load’ that increases chronic non-communicable disease risk, and the homeostatic ‘metabolic capacity’ that protects against these diseases, offers a new opportunity to improve assessment of disease risk. Importantly, this approach may improve the ability to take into account ethnic variability in chronic disease risk. This approach could be applied using simple measurements readily carried out in the home or community, making it ideal for M-health and E-health monitoring strategies.

## Introduction

For most of human history, the primary cause of morbidity and mortality was infectious disease. Life expectancy at birth averaged little more than three decades, and a large proportion of all those born died before reaching adulthood. Over the last two centuries, an increasing number of populations have undergone an epidemiological transition, characterized by demographic change associated with a decreased burden of infectious disease [1]. Consequently, the limiting factor for health and survival is increasingly the constitution of the body.

Globally, the leading cause of morbidity and mortality is now chronic non-communicable diseases, closely associated with the obesity epidemic, and the widespread adoption of unhealthy diets and behaviours such as smoking and physical inactivity [2–4]. In 2010, for example, ischaemic heart disease and stroke collectively killed one in four people worldwide, compared with one in five in 1990. Ischaemic heart disease is among the top four causes of death in every global region except Oceania and sub-Saharan Africa, and stroke is also one of the commonest causes of death in many regions. Already, 80% of the deaths from chronic diseases occur in low and middle-income countries, and a quarter occur in those below 60 years [2, 4].

This paper focuses on several chronic non-communicable diseases, namely hypertension, stroke, type II diabetes, and cardiovascular disease. Though these diseases affect different parts of the body, they have in common a generic life-course aetiology, as discussed below.

What kind of data can we use in order to (a) identify risk factors for these diseases, and (b) assess response to clinical management or public health interventions? We can search for such markers at many levels of biology: at the level of the gene, blood biochemistry, physiology, morphology, and behaviour. The challenge is that by seeking so many individual sources of information, we struggle to make sense of the complexity. What we need are simple proxies, suitable for widespread application, that provide reliable indications of relative disease risk.

The most obvious risk factor, demonstrated in large-scale epidemiological surveys, is excess body weight, most commonly expressed in the form of body mass index (weight divided by height squared, BMI). BMI can be relatively easily monitored within individuals through the life-course, although height may need to be re-measured from middle age onwards as it decreases slightly due to shrinkage. Given the close link between the epidemics of obesity and chronic diseases, BMI might appear a ‘panacea’ – the ideal trait for routine monitoring, and the best outcome for assessing the efficacy of public health interventions.

However, there is increasing dissatisfaction with BMI as a marker of chronic disease risk, for a number of reasons. First, within any population, there is substantial variability in the ratio of fat mass to lean mass at any given level of BMI, hence this outcome fails to reliably index any specific component of body composition [5, 6]. Second, again within populations, not all individuals develop health risks at the same BMI threshold. Some who are ‘overweight’ demonstrate metabolic perturbations, whereas others are ‘fat but fit’ [7], so that a high BMI may inadvertently flag metabolic ill-health in some who are actually healthy. Conversely, others may have metabolic risk despite their BMI lying in the normal range. Finally, between populations, there are systematic differences in the average level of body fat present at a given level of BMI [8–10].

More detailed measurements of body composition may offer a resolution to this scenario. Body composition reflects a wide variety of ‘levels’ of biology [11]. It is well established, for example, that body composition reflects the influences of genotype and gene expression [12–15]. However, the same traits also reflect patterns of development [16–20] as well as more immediate components of physiology such as glycemic control [21, 22]. Finally, body composition also relates to behaviour and parental care in early life [23–25] and current diet and activity level [26–30].

The aim of this paper is to briefly outline a conceptual model, demonstrating the potential utility of body composition data for indexing the risk of non-communicable diseases. Particular effort will be made to highlight how this approach may help address ethnic variability in chronic disease risk.

## The capacity-load model of disease risk

In the 1980s, chronic disease risk was widely attributed to two principal factors: current lifestyle, encapsulating factors such as unhealthy diet, obesity, smoking and physical activity, and genotype [31]. The importance of genetic factors was initially highlighted through family studies, showing the tendency for chronic diseases to cluster within families [32–34].

From the late 1980s a new perspective emerged, as studies repeatedly demonstrated that patterns of growth in early life also shaped chronic disease risk in adulthood. The pioneering work of David Barker and colleagues demonstrated consistent associations between low birth weight and chronic disease risk [35–39], with subsequent studies identifying independent contributions of rapid weight gain during childhood [40–42].

The first conceptual approach was developed by Hales and Barker [31], and was termed the ‘thrifty phenotype’ hypothesis. This model of disease assumed that the ability to resist the adverse metabolic consequences of unhealthy lifestyles in adulthood was undermined in those who had undergone poor growth in foetal life. It was suggested that low birth weight babies, experiencing foetal energy insufficiency, had sacrificed organs such as the pancreas in order to protect the brain [31, 43]. The result would be impaired glucose tolerance later in life, exacerbated on exposure to dietary richness. This approach initially led to the assumption that the long-term risks pertaining to low birth weight derived from some form of overt ‘under-nutrition’ during foetal life.

While this conceptual approach catalyzed the field, it gave undue emphasis to those with low birth weight. In fact, relevant data repeatedly showed that an inverse dose response association between birth weight and adult chronic disease risk was evident across the majority of the range of birth weight [37, 44–46], though for some outcomes disease risk increased again in those with the highest birth weights [47]. In other words, most chronic diseases in adulthood actually occur in those whose birth weight was within the normal range, and yet birth weight is still predictive of adult disease risk.

We therefore built on the thrifty phenotype hypothesis to develop an approach known as the ‘capacity-load’ model [48, 49]. This approach assumes that many components of adult lifestyle contribute to chronic disease risk. These include diet, physical inactivity, stress, smoking and air pollution, alcohol intake, as well as some effects of chronic infectious diseases. Collectively, all of these factors impose a ‘metabolic load’ that challenges the body's ability to maintain homoeostasis at the levels of cells, organs or tissues. The concept of metabolic load has much in common with that of allostatic load [50, 51], but instead of emphasizing the stress response, it highlights components of homoeostasis addressing fuel/lipoprotein metabolism and cardiovascular function.

The ability to tolerate this metabolic load is then considered to depend on traits, collectively termed ‘metabolic capacity’, that enable homeostasis to be maintained. Crucially, these traits develop during early ‘critical windows’ of development, meaning that they are strongly shaped by growth patterns in foetal life and infancy [48, 49]. Many specific physiological traits have been shown to scale relatively linearly with birth weight. Examples include nephron number in the kidney, neonatal lean mass, blood vessel caliber, airway size and metabolic functions such as insulin secretion. Broadly, the larger the size at birth, the greater the homeostatic capacity, though those with the highest birth weights may deviate from this pattern since much of their high weight is adipose tissue (metabolic load) rather than metabolic capacity. Consistent with the thrifty phenotype hypothesis, metabolic capacity is assumed to track from infancy into adulthood but may eventually deteriorate as part of the process of aging.

**Fig. 1.**
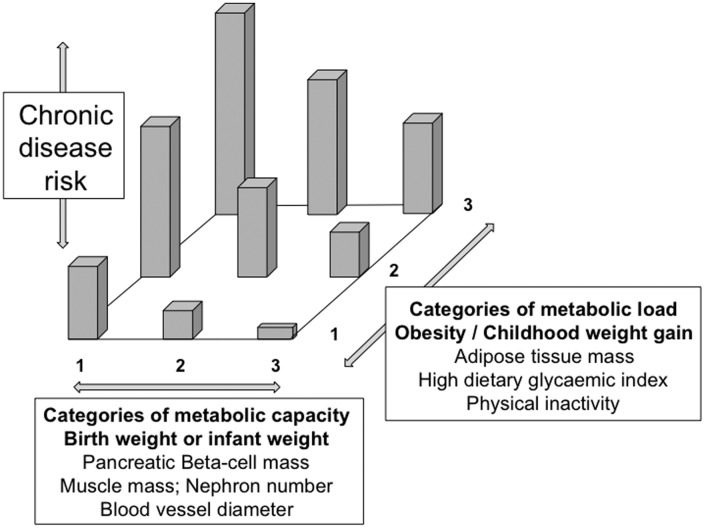
Schematic diagram of the ‘capacity-load’ model of chronic disease risk. Metabolic capacity promotes the maintenance of homoeostasis, and thereby reduces chronic disease risk. Metabolic load challenges homeostasis, and thereby elevates chronic disease risk. The highest risk of chronic disease is therefore found in those with high load and low capacity. Adapted and redrawn from ref 49.

**Fig. 2.**
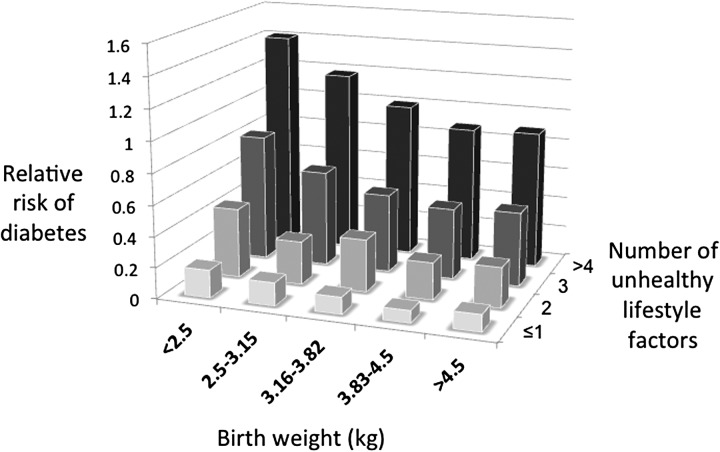
Empirical evidence supporting the capacity-load model of chronic disease risk for diabetes. The penalty for low birth weight steadily increases as the degree of unhealthy lifestyle increases. Based on data of Li *et al*. from 3 US cohorts [45].

The risk of chronic degenerative diseases can then be modelled as a function of metabolic load relative to metabolic capacity (Fig. 1). The highest risk of disease is anticipated in those with high metabolic load but low capacity [49], a scenario which has been demonstrated for numerous disease outcomes (Table 1) and is illustrated for diabetes risk in Fig. 2.

**Table 1.** Interactive associations between size at birth and subsequent weight in relation to chronic disease risk

Trait	Population	Reference
Blood pressure	Filipino adolescent boys	[108]
	Spanish youths	[109]
	Meta-analysis of 80 studies	[110]
Insulin resistance	Middle-aged Swedish men	[111]
	Indian children	[112]
	Meta-analysis of 48 studies	[113]
Glucose intolerance/diabetes	Finnish adults	[114]
	Indian adults	[41]
	Meta-analysis of 30 studies	[47]
LDL cholesterol	Middle-aged Swedish men	[111]
	Indian children	[112]
	Middle-aged Dutch adults	[115]
Triglycerides	Middle-aged Swedish men	[111]
	Middle-aged British adults	[116]
	Brazilian and Chilean adults	[117]
C-reactive protein	Finnish adults	[118]
	Black and white US adults	[119]
	Indian adults	[120]
Cardiovascular disease	Swedish adults	[121]
	Finnish adults	[42]
	Indian adults	[122]

For each outcome, lower birth weight (indexing reduced metabolic capacity) and higher BMI or adiposity (indexing metabolic load) independently increases disease risk. Reproduced with permission from ref 11.

Using this perspective, we can re-examine the utility of BMI as a marker of disease risk. BMI has been consistently associated with health and longevity in large populations, typically demonstrating a J shaped relationship [52, 53]. The thinnest groups have an elevated risk of mortality relative to those within the normal range, after which there is a dose response association with increasing morbidity and mortality. Recently, data have suggested that the ‘optimum’ BMI may be higher than previously assumed, such that the overweight may have the greatest longevity, but they may still have elevated chronic disease risk relative to the normal range [53, 54].

BMI is a very simple proxy for body composition, and its limitations as an index of adiposity are well established [5], so why should it be able to index broader patterns of health status and disease risk? We have previously suggested that the utility of BMI derives from it indexing both current weight (metabolic load), and completed growth (height, associated with birth weight and hence metabolic capacity) [55]. For example, numerous studies have linked short stature with an increased risk of chronic diseases [56–60]. A high BMI value therefore provides a very simple index of capacity-load status.

However, the utility of BMI is much less impressive when we focus on individuals, and particularly when they belong to different ethnic groups. It is now clear that the association between BMI and chronic disease risk is confounded by ethnic differences in size, physique and adiposity. For example, Indians develop diabetes following relatively modest increments in BMI through young adulthood [41]. More detailed indices of body composition could therefore help resolve this scenario, by providing independent proxies for each of metabolic capacity and metabolic load.

## Body composition and metabolic load

In terms of body composition, the most obvious component of metabolic load may be total fat mass. However, there is increasing recognition that the regional anatomical distribution of body fat also affects metabolic profile. Studies have repeatedly demonstrated that central abdominal fat, in particular visceral fat, is metabolically more harmful than peripheral fat in the gluteo-femoral region [61–64]. For this reason, indices of adiposity that take into account its regional distribution may be more successful in predicting chronic disease risk.

A large study demonstrated that waist-hip ratio was more successful than BMI at predicting cardiovascular mortality across 52 countries [65]. Mortality was much greater in those with high waist girth but low BMI, compared with those with high BMI but low waist girth. Abdominal fat correlates with many components of the metabolic syndrome, including elevated fasting glucose and insulin levels, cholesterol levels, blood pressure, and inflammatory markers. Indeed, obesity manifests as a chronic inflammatory state [66, 67]. It is also widely recognized that low levels of physical activity increase the risk of obesity, while unhealthy diets (high in processed sugar) are also correlated. Both of these factors are independent components of unhealthy metabolism and predict mortality [68–70].

We should not therefore be surprised that indices of adiposity are very valuable markers of chronic disease risk, by indexing metabolic load. This has been confirmed by extensive data indicating that the global obesity epidemic is a strong environmental factor driving the chronic disease epidemic [2–4].

However, measurements of adiposity may still require ethnic differences to be taken into account. It is already recognized that populations differ in their body fat content for a given BMI value. For example, Asian populations tend to have elevated body fat, and African or Caribbean populations lower levels of body fat, for a given BMI value compared with European populations [8–10, 71]. This means that the threshold at which body weight becomes unhealthy is expected to differ across populations. An effort to resolve this has resulted in ethnic specific BMI cut-offs for defining overweight and obesity [72].

Direct measurements of body fat and body shape could overcome some of these limitations, especially as the regional distribution of body fat also differs between ethnic groups. Furthermore, some studies suggest that the metabolic toxicity of body fat varies between ethnic groups. For example, the association between body fat and insulin resistance was stronger in South Asian compared with European and African and Caribbean children in the UK [73].

This indicates that body composition can provide a very valuable index of metabolic load, though it may still be difficult to compare different ethnic groups on a common basis. There are now a number of techniques available for collecting body composition data, including DXA, air-displacement plethysmography, and magnetic resonance imaging [74]. For widespread routine monitoring, waist girth remains the simplest option, though there is uncertainty as to whether it should be indexed to height or to hip girth [75], or simply expressed in absolute units. One potentially exciting opportunity is the development of 3-D photonic scanning of body shape [76]. This non-invasive method provides a rapid but detailed assessment of physique, though not of internal tissues. It is ideal for monitoring body shape changes, and has already been used in large ‘sizing surveys’ for the clothing industry [77–79]. With the instrumentation suitable for use in health clubs, shopping malls and clinics, it may prove to be a valuable means of monitoring metabolic load.

Another component of metabolic load relatively easily measured is physical inactivity, through the use of pedometers, or accelerometers worn on the waist or wrist. These could be readily adapted to download data to central digital data collection points.

## Body composition and metabolic capacity

Where birth weight data are available, it is now clear that they provide valuable information on chronic disease risk. For example, in a study of Swedish adults, Leon *et al*. [80] showed that the metabolic penalties for tall height and obesity occurred primarily in those of low birth weight. In other words, the extent to which metabolic load increases disease risk is strongly shaped by metabolic capacity.

Recent studies have linked birth weight with more detailed structural and functional components of the cardiovascular system. These associations are evident across a wide age-span, indicating that they emerge early in life and then track subsequently. Relevant outcomes include endothelial function, aortic size and wall thickness, aortic root diameter and vascular mechanical properties of other integral arteries.

In infants, children and adolescents, for example, birth weight has been inversely associated with several measures of cardiac competence (Table 2). Skilton *et al*. [81] reported a significant, negative relationship between birth weight and thickening of the aortic wall in 25 growth-retarded neonates when compared with those of normal birth size. Another study reported smaller vessel diameters in the abdominal aorta, popliteal artery, and common carotid artery in adolescents born small for gestational age [82]. These and other vascular properties affect cardiac load and the regulation of blood pressure, and imply an increased risk of cardiovascular complications.

**Table 2.** Birth weight associated with cardiac outcomes in children/adolescents

Sample	Outcome	Gestational age profile	Association/Findings	Reference
115 boys and 101 girls aged 9 years from Southampton, UK	Total coronary artery diameter; aortic root diameter; left ventricular outflow tract diameter	Mean birth weight 3.37 kg for boys, 3.26 kg for girls. 7.8% of boys and 6.9% of girls born premature. Results similar after adjustment for gestational age	Increase in: coronary artery diameter by 0.10 mm (95% CI 0.03–0.16), log aortic root diameter by 1.5% (95% CI 0.1–2.8%), and log left ventricular outflow tract diameter by 1.6% (95% CI 0.5–2.6%) per s.d. increase in birth weight	[123]
400 newborns admitted to the Women's Hospital of Los Angeles County, USC Medical Center, USA	Aortic root diameter	Birth weight ranged from 750–4750 g and gestational age from 25–43 weeks. 172 preterm and 228 term-born.	Aortic root diameter increased linearly with increase in birth weight, and also with increasing gestational age	[124]
1369 children aged 6 years from the Sydney Childhood Eye Study, Sydney, Australia	Retinal microvascular caliber	23 children of very low birth weight (<2000 g), 60 low birth weight (2000–2499 g) and 1286 of normal to high birth weight (>2500 g). 112 born preterm	Retinal arteriolar caliber narrowed by 2.25 um (95% CI 0.57–3.92, *p* = 0.01) per 1 kg decrease in birth weight, demonstrating a gradient effect	[85]
24 young women and 20 young men, mean age 17.5 years from prospective cohorts in Malmö, Sweden	Vascular mechanical properties of the common carotid artery, the abdominal aorta and popliteal artery	21 born with IUGR (birth weight ≥2.5 s.d. below mean weight of the normal population) and abnormal fetal aortic blood flow; gestational age 270 (s.d. 13) days. 23 born AGA at 278 (s.d. 9) days	The IUGR group had significantly smaller end-diastolic vessel diameters in the abdominal aorta and popliteal artery; similar but non-significant trend in common carotid artery	[82]
86 healthy adolescents aged 15 years from the Stockholm Neonatal Project, Sweden	Aortic size	45 born preterm with an average gestational age of 28 weeks and birth weight <1500 g. 41 controls born at term.	Subjects born preterm had significantly narrower aortic lumen after adjustment for confounders	[125]
50 newborns at the Royal Prince Alfred Hospital, Sydney, Australia	Aortic wall thickness	25 newborns with IUGR (gestational age 38–40). 25 with normal birth weight (gestational age 39–41).	Significant aortic wall thickening in IUGR group compared with normal birth weight group after adjustment for confounders	[81]
39 young adults aged 19–21 from the Cardiff Births Survey, UK	Endothelial function (by flow-mediated dilatation)	22 low birth weight (<2.5 kg) and 17 normal birth weight (3.0–3.8 kg). All born at ≥38 weeks gestation	Flow-mediated dilatation was significantly impaired in low birth weight group relative to a group with normal birth weight	[126]
165 British girls and 168 British boys, aged 9–11	Endothelial function (by flow-mediated dilatation)	No information on gestational age	Significant, positive, graded association between birth weight and flow-mediated dilatation	[127]
35 girls and 43 boys aged 8–13 in São Paulo, Brazil	Endothelial function (by flow-mediated dilatation)	Normal birth weight group (*n* = 36) born at term with birth weight ≥3.0 kg. Low birth weight group (*n* = 42) born at term but SGA, with birth weight ≤2.5 kg.	Significant, positive, graded association between birth weight and flow-mediated dilatation	[128]
21 boys and 23 girls, aged 7–11 born in Danderyd Hospital, Stockholm, Sweden	Carotid artery stiffness	22 subjects reported low birth weight (<2500 g) for age. All born at term	Significant, negative correlation between birth weight and stiffness of the carotid artery wall	[129]

Investigations in adults show similar findings (Table 3). Low birth weight was associated with narrower retinal arteriolar caliber (a marker of hypertension and cardiovascular disease risk) among 3800 individuals aged 51–72 years [83]. In a Dutch cohort, birth weight was inversely associated with carotid intima media thickness (CIMT), indicative of subclinical atherosclerosis, in the lowest tertile of birth length [84]. Additionally, birth weight was inversely associated with CIMT in subjects demonstrating ‘catch-up’ growth in infancy, another risk factor for adult chronic disease and mortality [40]. However, it should be noted that links between early-life and cardiovascular outcomes in adults may be confounded by other conditions such as diabetes and hypertension that may reflect both developmental and current lifestyle influences [85].

**Table 3.** Birth weight associated with cardiac outcomes in adults

Sample	Outcome	Gestational age profile	Association/Findings	Reference
3800 adults aged 51–72 from the Atherosclerosis Risk in Communities Study, USA	Retinal arteriolar calibre	All term-born	Significant, positive, graded association between lower birth weight and narrower retinal arteriolar caliber, 161.0 um for <2.5 kg *v.* 163.1 um for ≥4.0 kg, *p* = 0.005	[83]
296 men and women born in Sheffield, UK, aged 50–53	Arterial compliance (by pulse wave velocity)	All term-born	Significant, negative, graded relationship between birth weight and pulse wave velocity in the femoro-popliteal-tibial arterial segment indicate decreased arterial compliance in the legs and trunk	[130]
125 men and 61 women born in Sheffield, UK, aged around 70 years	Atherosclerosis in the carotid and lower limb arteries	Sample included some pre-term-born individuals, but a separate analysis excluding those born before 37 weeks is reported	Significant, graded increase in risk of carotid atherosclerosis as birth weight decreases; odds ratio for atherosclerotic disease in the lower limbs highest in those with lower birth weight, but non-significant	[131]
150 men and 165 women aged 20–28 from Cambridge, UK	Endothelial function (by flow-mediated dilatation)	No information on gestational age	Graded effect of birth weight on vascular function significant and adverse, but the addition of acquired risk factors to the model ‘overwhelmed the association’	[132]
352 men and 398 women aged 27–30 from the Atherosclerosis Risk in Young Adults (ARYA) study, the Netherlands	Subclinical atherosclerosis measured as carotid intima media thickness (CIMT)	Mean gestational age 39.8 (s.d. 1.8) for men, 39.8 (s.d. 2.0) for women; 32 subjects born premature, but results shown with these individuals excluded	Significant, negative association between birth weight and CIMT in the lowest tertile of birth length only: −12 um/kg (95% CI −6 to −18). Also, an inverse association was found between birth weight and CIMT in those with exaggerated infant growth: −35 um/kg (95% CI −18 to −52)	[84]

The main limitation of birth weight as a marker of metabolic capacity is that the information may not be available for many individuals, especially from low- and middle-income populations. However, other proxies can be used in its place.

Some aspects of metabolic capacity may be indexed by childhood growth patterns. It is now clear that poor childhood growth impacts the lower leg in particular, resulting in shorter legs relative to total height [86, 87]. A number of studies have demonstrated elevated chronic disease risk in those with shorter leg length in adult life [58, 88–90].

Of particular interest, relative leg length (i.e. leg length/height) appears minimally correlated with birth weight, meaning that measurement of this trait in adult life provides an assessment of postnatal as opposed to foetal growth [86, 91]. Some aspects of metabolic capacity, such as the pancreas, appear to continue to develop during postnatal life [92], potentially explaining why short leg length is an independent risk factor for diabetes. Relative leg length is therefore subtly different from leg length *per se*, by being independent of birth weight, and the two traits may potentially be used in combination to index metabolic capacity when data on birth weight are unavailable.

However, a cautionary note is necessary. A Swedish study showed that tall stature may also elevate disease risk in those born small [80]. This suggests that compensatory catch-up growth occurred after birth in this group, reducing the utility of height as a marker of metabolic capacity. As yet, it is unclear if this issue could be resolved by focusing in more detail on leg length or relative leg length.

Until recently, very few other simple options were available for assessing metabolic capacity in adult life. One approach is the prediction of lean mass using bio-electrical impedance analysis. However, even ignoring the relatively poor precision of this approach at the level of the individual, the association of total body lean mass with health also appears complex. On the one hand, lean mass incorporates muscle mass, which is widely considered to protect against diabetes. On the other hand, some studies have linked high levels of lean mass with higher blood pressure [90, 93]. Total lean mass may therefore be too generalized to act as a reliable proxy for metabolic capacity in adult life.

Recently, much attention has been paid to a more specific component of body composition, measured at the level of function rather than mass. Grip strength, often considered a marker of muscle strength, has attracted interest because it is positively associated with cardio-metabolic function in children (e.g. [94]), and negatively related to morbidity and mortality in adults [95–100]. Like BMI, grip strength may provide a valuable proxy for several different traits, each of which is associated with chronic disease risk. We therefore review this new opportunity for indexing metabolic capacity in more detail.

## Grip strength as a potential marker of metabolic capacity

**Fig. 3.**
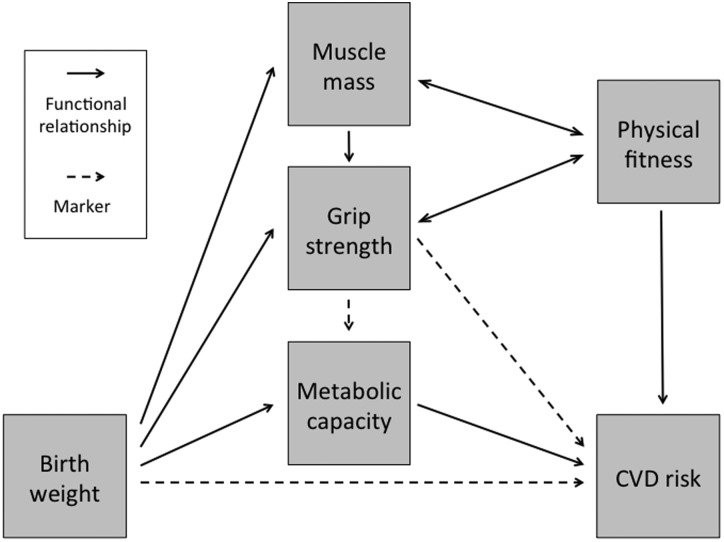
Schematic diagram illustrating how grip strength may act as a valuable marker of chronic disease risk through its ability to index two crucial components of metabolic capacity: foetal growth (its development) and adult physical fitness (its maintenance).

What is particularly valuable about grip strength is that it may simultaneously index both the early-life development of metabolic capacity, as well as reflecting current physical fitness, which is also important for health. Birth weight has been repeatedly associated both with lean mass [101] and with grip strength, as discussed below. Looking in the reverse direction, grip strength therefore reflects foetal growth experience, and may act as a marker of metabolic capacity. Beyond this, grip strength also reflects current lifestyle, with those currently physically active likely to have greater physical fitness. This conceptual approach is summarized in Fig. 3.

A number of authors have reported significant associations between birth weight and adult grip strength (Table 4). A recent meta-analysis found a 0.86 kg (95% CI 0.58–1.15) increase in grip strength per kg increase in birth weight in gender-pooled data from 13 studies [113]. A number of the studies included in this meta-analysis are also included in Table 4. Variation in the reported B-coefficients is potentially due to several factors, including variation in subject age, methods of hand grip measurement, gender, and variable adjustment for potential confounders, but all studies show a significant positive association with the exception of Patel *et al*. [103], whose trend did not reach significance.

**Table 4.** Birth weight associated with adult grip strength

Sample	Findings	Gestational age profile	Reference
Meta-analysis of 19 studies Pooled-gender results calculated for 13 studies with sufficient data	Increase in grip strength of 0.86 kg (95% CI 0.58–1.15) per kg increase in birth weight	No discussion of gestational age	[133]
1562 women aged 20–40 years from the Southampton Women's Survey, UK. Grip strength measured at 19-weeks pregnancy.	Increase in grip strength of 2.16 kg (95% CI 1.62–2.70) per kg increase in birth weight	No information on gestational age. Mean birth weight 3.24 (s.d. 0.56) kg	[134]
1371 men and 1404 women aged 53 years from the MRC National Research Survey of Health & Development, a prospective national birth cohort in the UK	Increase in grip strength of 1.83 kg (95% CI 0.66–3.01) for men and 1.27 kg (95% CI 0.45–2.10) in women per kg increase in birth weight	Mean birth weight 3.5 (s.d. 0.5) in each sex. No information on gestational age	[135]
105 men aged 68–76 years from the Hertfordshire Cohort Study, UK	Non-significant trend for men in the low birth weight group to have lower grip strength	Birth weight <3.18 and >3.63 kg	[103]
2071 men and 2233 women aged 31 years from the 1966 Northern Finland Birth Cohort	Increase in grip strength of 1.42 kg (95% CI 1.19–1.65) per s.d. increase in birth weight, adjusting for sex and gestational age; equivalent to increase in grip strength of 3.0 kg per kg increase in birth weight	Mean birth weight 3.60 kg (s.d. 0.50) for men, 3.47 kg (s.d. 0.47) for women. Analysis restricted to singleton infants born at ≥36 weeks gestation	[136]
1569 men and 1414 women aged 59–73 years from the Hertfordshire Cohort Study, UK	Increase in grip strength of 2.06 kg (95% CI 1.38–2.74) in men and 1.50 kg (95% CI 0.91–2.10) in women per kg increase in birth weight	Mean birth weight 3.50 kg (s.d. 0.54) for men, 3.35 kg (s.d. 0.50) for women. No information on gestational age at birth	[137]
411 men and 306 women, average age 67.5 years, born in Hertfordshire, UK	Grip strength correlated significantly with birth weight (*p* < 0.01)	Mean birth weight 3.53 kg (s.d. 0.50) for men, 3.41 kg (s.d. 0.47) for women. Gestational age was adjusted for	[138]
928 men and 1075 women aged 56–70 years born at Helsinki University Central Hospital	Increase in grip strength of 1.84 kg (95% CI 0.62–3.06) in men and 1.79 kg (95% CI 0.94–2.64) in women per kg increase in birth weight. These associations were attenuated with adjustment for age and adult BMI	Mean birth weight 3.48 kg (s.d. 0.50) for men, 3.35 kg (s.d. 0.47) for women. Gestational age data available for 1880 individuals, all reported term-born	[139]

The utility of grip strength for predicting chronic disease outcomes was recently demonstrated by Leong *et al*. [100]. In a large, multi-ethnic and socioeconomically variable sample followed over 4 years, these authors showed inverse associations between grip strength and all-cause mortality, cardiovascular mortality, non-cardiovascular mortality, myocardial infarction, and stroke. Indeed, grip strength was a stronger predictor of all-cause and cardiovascular mortality than systolic blood pressure. This study thus highlights the potential for grip strength to assess chronic disease risk in individuals for whom overt symptoms are not yet evident, and for whom information on birth weight is not available.

## A composite capacity-load model

We therefore propose an enhanced version of the ‘capacity-load’ model, for application in the assessment of chronic disease risk in populations where data on birth weight are lacking. Metabolic load can be categorised by a combination of BMI, waist girth, and physical inactivity. For example, a clustered *z*-score (the average of several raw z-scores, as already used for the assessment of metabolic risk in children [104]) may be calculated based on raw data for these variables. Metabolic capacity may be categorised through grip strength, leg length and relative leg length, again using a clustered z-score approach. We then assume that chronic disease risk is greatest in those with a high ratio of metabolic load to metabolic capacity.

This approach is consistent with recent work intended to improve the assessment of sarcopenic obesity, where high levels of body fatness coexist with unhealthily low levels of lean tissue mass [105]. This condition is increasingly prevalent, and is considered a key pathway linking body composition with metabolic ill health. We have recently published capacity-load centile charts for sarcopenic obesity based on adult body composition, namely the ratio of fat mass to fat-free mass, and the ratio of trunk fat to appendicular skeletal muscle mass [106]. This approach could therefore be extended as described above, to incorporate clustered scores of metabolic capacity and load.

## Strengths and limitations

There is of course no panacea for assessing chronic disease risk in public health research and practice. No single trait can reliably index health risk in all individuals, or accurately summarize the beneficial responses to public health interventions. A limitation of our approach is that while data on early life growth and current body composition may surpass BMI at indexing chronic disease risk, they still may lack the sensitivity of physiological outcomes such as blood pressure or blood biochemistry. Moreover, sophisticated body composition measurements do not inevitably outperform BMI. In 2369 adults from Hyderabad in India, for example, waist-hip ratio was only slightly better than whole-body adiposity at predicting diabetes risk, and BMI performed as well as adiposity in predicting other markers of cardiovascular risk [107].

Nevertheless, findings such as those illustrated in Fig. 2 suggest that integrating data on experience in early life and current phenotype should improve chronic disease risk assessment over measures of adult phenotype alone. Our hypothesis is that accurate measurements of load will categorize risk best when combined with accurate measurements of capacity.

A potential strength of our proposed approach is that it may prove adequate for monitoring changes over time, without the need for expensive or intrusive tests. Indeed, individuals may monitor most outcomes themselves in the community, offering the potential to link with M-health and E-health monitoring. Baseline measurements of leg length could be collected, while subjects could then monitor their weight, activity level using pedometry, waist girth, and grip strength.

This approach merits testing in large cohorts to establish its sensitivity for estimating chronic disease risk and mortality risk. Changes in weight, waist girth, and physical activity may be relatively sensitive to dietary shifts, which are relatively hard to quantify directly with accuracy. The recent demonstration that grip strength proved capable of indexing ethnic differences in chronic disease risk was particularly informative and encouraging.
